# Induced Hypotension in Functional Endoscopic Sinus Surgery: A Comparative Study of Dexmedetomidine and Esmolol

**DOI:** 10.7759/cureus.15069

**Published:** 2021-05-17

**Authors:** Baladev P Sahu, Laba K Nayak, Partha S Mohapatra, Krishna Mishra

**Affiliations:** 1 Department of Anaesthesiology, Kalinga Institute of Medical Sciences, Bhubaneswar, IND; 2 Department of Community Medicine, Kalinga Institute of Medical Sciences, Bhubaneswar, IND

**Keywords:** hypotensive anesthesia, esmolol, dexmedetomidine, emergence time, sedation score, rebound hypertension, postoperative analgesic demand, fess, hemodynamic stability

## Abstract

Introduction

Functional endoscopic sinus surgery (FESS) is one of the common surgical procedures requiring hypotensive anesthesia; many agents have been tried to reduce the amount of blood loss. This study aims at comparing the efficacy of two agents for providing deliberate hypotension.

Objectives

The aim of this study was to evaluate the efficacy of esmolol and of dexmedetomidine and compare which one of the two is a better agent to produce induced hypotension during FESS.

Materials and methods

This was a comparative study conducted in a tertiary care hospital in Odisha, India. There were two study groups with 30 participants each who were given either esmolol or dexmedetomidine (group E and group DEX, respectively).

Results

Esmolol is an anti-hypertensive agent with better hemodynamic stability. The amount of drug and dose requirement was low in group DEX. The emergence time, sedation score, and time to first analgesic request were found to be highly statistically significant in group DEX.

Conclusion

Dexmedetomidine was found to be a better agent at controlling intra-operative blood pressure than esmolol and had beneficial effects on recovery from anesthesia and analgesia.

## Introduction

All surgical incisions involve cutting of the blood vessels, where simple persistent oozing may add much difficulty to even the simplest surgical procedures while massive bleeding can be hazardous and require transfusion. Deliberately induced hypotension is a method to produce a controlled and safe reduction in the arterial blood pressure (BP) while preserving organ perfusion by reducing the BP of the patient by almost 20% by decreasing capillary ooze [1]. It is the prime concern of every ENT surgeon to delineate the intricate anatomy and thus the need for a bloodless, clear operative field, which is obtained by induced systemic hypotension. Various drugs are used such as esmolol, which is an ultra-short-acting selective B1 adrenergic antagonist that decreases heart rate (HR) and BP with a rapid onset of action [2]. Dexmedetomidine can also be used to prevent tachycardia and hypertension; it is a highly selective α2 adren­ergic receptor agonist with sympatholytic, anti-anxiety, and sedative and analgesic effects [3]. With the above considerations, this study aimed to compare the efficacy of esmolol and dexmedetomidine in achieving hypotensive anesthesia during functional endoscopic sinus surgery (FESS).

## Materials and methods

The aim of this study is to compare the efficacy of dexmedetomidine with that of esmolol in successfully providing induced hypotension in FESS.

It was an experimental prospective comparative study to compare the efficacy of hypotensive anesthesia achieved by using esmolol and dexmedetomidine during FESS and was undertaken in the Department of Anesthesiology and Critical Care at SCB Medical College and Hospital, Odisha, India. The study was conducted from September 2015 to October 2017 and included 60 study participants who were randomly selected from the Department of ENT who were planned for elective FESS. Two drugs capable of inducing hypotension, namely esmolol and dexmedetomidine, were considered for the surgery, and each group had 30 study participants with random allocation. Group E was the group with patients who received intravenous (IV) esmolol infusion, and group DEX comprised patients who received IV dexmedetomidine infusion. The study was initiated after getting due permission from the Hospital Ethical Committee. Written informed consent was obtained from all the patients undergoing FESS.

Inclusion criteria comprised patients of both the sexes in the age group of 25-50 years, only those patients belonging to ASA (American Society of Anaesthesiologists) grades I and II, and those planned for elective FESS in the ENT operation theater under general anesthesia (GA).

Exclusion criteria comprised patients with hypertension and coronary artery disease, patients with renal, hepatic, or cerebral insufficiency, patients with coagulopathy or receiving drugs affecting coagulation, patients with addiction to alcohol, smoking, and narcotics, grossly anemic and hypovolemic patients, chronically diseased and debilitated patients, patients aged more than 50 years, patients not giving written informed consent, patients of ASA grade III, IV, or V, and patients with significant baseline bradycardia.

The preoperative assessment of all the patients was conducted by history taking, detailed clinical examination, systemic examination, and essential laboratory investigations.

The specific examination involved assessment of the upper airway anatomy and difficulty in intubation using the Mallampati classification; patients who had a score of more than grade II were excluded [4]. Cardiorespiratory reserve was assessed using the Sabrasez test, and breath-holding for more than 30 seconds was considered as fitness for GA [5].

Methodology

All the cases planned for FESS were kept on overnight fasting and tablet pantoprazole and tablet alprazolam 0.5 mg was given orally on the night before operation. Infusion of Ringer’s lactate solution was administered at the rate of 8-10 mL/kg/hour to maintain an hourly urine output of at least 1 mL/kg/hour (50 mL or more) and adjusted as per fasting status. In the operating theater, a preoperative check of BP, pulse rate, and SpO_2_ was done, and electrocardiogram (ECG) cables were attached for monitoring. In group DEX, patients received a loading dose of 1 μg/kg of dexmedetomidine diluted in 10 mL of 0.9% normal saline infused over 10 minutes before induction of anesthesia followed by continuous infusion of 0.4-0.8 μg/kg/hour. In group E, patients received 1 mg/kg of esmolol as a loading dose infused over 1 minute followed by continuous infusion of 0.4-0.8 mg/kg/hour. In both the groups, the infusion was titrated to maintain mean arterial pressure (MAP) within 55-65 mmHg. The patients were pre-medicated with injection glycopyrrolate 0.2 mg IV, injection midazolam 0.05 mg/kg IV, and injection nalbuphine 0.03 mg/kg 30 minutes prior to induction. The radial artery was cannulized using a 20G cannula connected to a transducer for beat-by-beat BP monitoring. A central line with 7-French triple lumen catheter was introduced to the right internal jugular vein for central venous pressure (CVP) monitoring. All the patients were catheterized with Foley’s catheter for measuring urine output. Patients were pre-oxygenated for three minutes with 100% oxygen at a flow rate of 6 L/min. All the patients were induced with an induction dose of propofol at 2 mg/kg IV. All the patients were intubated with appropriate size cuffed endotracheal tube after adequate relaxation by injection vecuronium 0.1 mg/kg IV and mask ventilation with 100% oxygen. The cuff was inflated just to obliterate audible leakage and fixed with adhesive tape. Anesthesia was maintained with 66% N_2_O in oxygen and 0.8 vol% isoflurane, and adequate muscle relaxation was achieved with intermittent vecuronium bromide. Ventilation (tidal volume of 6-8 mL/kg) was adjusted to maintain end-tidal carbon dioxide (EtCO_2_) at <35 mmHg. Monitoring of the parameters such as BP, HR, SpO_2_, EtCO_2_, ECG, CVP, temperature, and urine output was done. The SBP (systolic blood pressure) was lowered to less than 100 mmHg before the start of surgery and maintained in the range of 80-100 mmHg throughout the surgery in both group E and group DEX by adjusting the dose of the drugs. All the data were recorded on a specially prepared anesthesia record sheet.

For evaluation of the visibility of the operative field during surgery, the average category scale was used as used in many studies [6]. Grade 1 comprised cases with slight bleeding where no suctioning of blood was required, grade 2 comprised cases with slight bleeding where occasional suctioning was required and surgical field was not threatened, Grade 3 comprised cases with slight bleeding requiring frequent suctioning and a threatened surgical field a few seconds after suction was removed, grade 4 comprised cases with moderate bleeding requiring frequent suctioning and bleeding threatened surgical field directly after suction was removed, and grade 5 comprised cases with severe bleeding requiring constant suctioning. Bleeding appeared faster than could be removed by suction, and the surgical field was threatened severely making the surgery impossible.

The hypotensive agents were discontinued 10 minutes before the end of surgery. The SBP was allowed to return to pre-hypotensive levels or near it to check for hemostasis. The operative time was measured from the start of skin incision to the end of skin closure. Fluid input during surgery was determined based on preoperative fasting, blood loss, and clinical criteria (arterial pressure, HR, and observation of the patient). Injection ondansetron 8 mg IV was given at the end of surgery to control nausea and vomiting. The patients were reversed at the end of surgery with appropriate doses of injection neostigmine (0.05 mg /kg) and injection glycopyrrolate (0.01 mg/kg). The patients were constantly monitored for vital functions such as NIBP (non-invasive blood pressure), SBP, DBP (diastolic blood pressure), MAP, HR, and urine output from premedication till the recovery and recorded as before premedication, before induction, before intubation, after intubation, every 5 minutes till 30 minutes, and then every 10 minutes till 90 minutes of the start of the hypotensive agent. Emergence time defined as the interval between the discontinuation of anesthetics and response of eye opening to verbal command was recorded. After extubation and full recovery, the patients were transferred to the post-anesthetic care unit to be observed where time to the first analgesic requirement was recorded. Postoperative recovery was evaluated using a modified Aldrete score [7,8]. Score (0-10) and time needed to achieve a score of greater than or equal to 9 were recorded. Sedation score was measured using the Ramsay sedation scale at 15, 30, and 60 minutes after tracheal extubation [9].

A sedation score of 1 was for patients who were anxious and agitated or restless or both, score 2 was for cooperative, oriented, and tranquil patients, score 3 was for those responding to commands only, score 4 was for brisk response to a light glabellar tap or loud auditory stimulus, score 5 was for sluggish response to a light glabellar tap or loud auditory stimulus, and score 6 was for those with no response to a light glabellar tap or loud auditory stimulus.

Effectivity of the hypotensive technique was judged, taking into account the mean intra-operative blood loss, number of patients requiring intra-operative blood transfusion, surgeon’s score (about the dryness of surgical field), emergence time (in minutes), time (in minutes) to modified Aldrete score > 9, sedation score 15 minutes after surgery, sedation score 30 minutes after surgery, sedation score 60 minutes after surgery, and time to first analgesic request (in minutes).

Data were entered into a Microsoft Excel sheet, and SPSS Version 22 (IBM Corp., Armonk, NY) was used for the analysis. The data were expressed as mean and standard deviation. Analysis of variance was used to find the homogeneity of baseline characteristics between two groups of patients. Analysis of variance was also used to find the significance of hemodynamics between two groups of patients. Tests used were the chi-square test, unpaired Student’s t-test, analysis of variance, and non-parametric comparison of median values of the two groups whenever required. A p-value of <0.05 was considered to be statistically significant.

## Results

A total of 60 patients were included in the study and were grouped into group E and group DEX depending on the anesthetic agent used for FESS. Table 1 shows the distribution of study participants according to demographic features.

**Table 1:** Distribution of the study participants according to demographic features E, esmolol; DEX, dexmedetomidine

	Group				Total		Chi-square, df, and p-value
	E		DEX				
	n	%	n	%	n	%	
Age group (in years)							
25-30	10	33.33	11	36.67	21	35	X^2^=1.586, df=3, p=0.663
31-40	15	50.0	14	46.67	29	48.39	
41-50	5	16.67	5	16.67	10	16.67	
Gender							
Male	26	88.67	24	80	50	83.33	X^2^=0.480, df=1, p=0.488
Female	4	13.33	6	20	10	16.67	
Weight group (in kg)							
35-50	12	40.00	9	30.00	21	35.00	X​​​​​​​^2^=0.669, df=1, p= 0.417
51-65	18	60.00	21	70.00	39	65.00	

Table 1 shows that majority of the study participants were in the age group of 31-40 years and most of them were males. The majority of the participants weighed around 51-65 kg. The socio-demographic characteristics of the study participants in both the groups did not have any statistical significance with the type of anesthetic agent used in FESS.

Table 2 shows the preoperative parameters of the study participants.

**Table 2:** Comparison of preoperative parameters of the study participants HR, heart rate; SBP, systolic blood pressure; DBP, diastolic blood pressure; MAP, mean arterial pressure

	Mean ± SD		t	p-Value
	Group E	Group DEX		
Pre-operative HR (beats per minute)	93.133 ± 4.167	91.5 ± 7.262	1.068	0.290
Pre-operative SBP (mmHg)	123.267 ± 7.547	124 ± 5.62	-0.466	0.643
Pre-operative DBP (mmHg)	79.8 ± 4.475	80.533 ± 3.998	-0.669	0.506
Pre-operative MAP (mmHg)	94.333 ± 5.3	95.133 ± 4.167	-0.650	0.518

Table 2 clearly reveals that preoperative HR, SBP, DBP, and MAP are more or less equal in both the study groups. The equality of preoperative vital parameters was studied using the independent sample t-test, which revealed no statistical significance in the values of the respective parameters between the two study groups. Thus, the pre-requisite for validity of results is satisfied.

In group E, the mean duration of surgery was found to be 88.27±10.031 minutes and in group DEX it was found to be 90.833±10.158 minutes. The mean values between the two groups are comparable (p=0.193). In the present study, the vital parameters were recorded till 90 minutes, the initial 30 minutes at an interval of 5 minutes, and thereafter at an interval of 10 minutes.

On comparing the duration hypotensive anesthesia, it was found that in group E (66.667±11.345 minutes) and group DEX (64.223±12.071 minutes), there was no statistical significance (p=0.424). The mean time taken for the onset of hypotension in group E (10.867±1.456 minutes) and group DEX (11.2±1.846 minutes), and there was no statistically significant difference (p=0.441).

Mean DBP at the start of the agent was little lower than that of intubation, but there was no difference between the two groups (p=0.822). Time series plot of DBP revealed that in both groups, there was a steady fall in the DBP till 15 minutes and was maintained at that level till 60 minutes, i.e., DBP was maintained at a constant level throughout the entire intra-operative period. After the stoppage of the hypotensive agent, there was a steady rise in DBP that reached to the initial level, but mean DBP in group DEX was slightly lower than that in group E. This implied that the hemodynamic changes reverted to normal faster in group E than group DEX, emphasizing the fact that in case of surgeries requiring longer time interval, dexmedetomidine is a better agent for induced hypotension due to delayed surgical site perfusion. Similar observation was for SBP and MAP, where there was a steady rise that reached to the initial level, but rise in group E was faster than in group DEX, which represented a rapid perfusion of the surgical field following anesthetic agent withdrawal, which is not preferred in FESS. After stoppage of the hypotensive agent, the mean HR in group E was slightly higher than that in group DEX.

The dose required for the onset of hypotension in group E was 0.489±0.014 mg/kg, whereas it was 1.016±0.056 µg/kg in group DEX. This showed that the amount of anesthetic agent required to induce hypotensive anesthesia in Group DEX was significantly low than the other group. This difference was found to be highly statistically significant. The total amount of drug consumed to produce hypotensive anesthesia in group E was 1.6±0.179 mg/kg, whereas it was 1.8±0.146 µg/kg in group DEX, and this difference was found to be highly statistically significant.

Table 3 shows the recovery statistics of the study participants.

**Table 3:** Recovery characteristics, sedation scores, and time to first analgesic request (n=60)

	Mean ± SD		t	p-Value
	Esmolol	Dexmedetomidine		
Emergence time (minutes)	4.933 ± 0.58	8.287 ± 0.645	-21.164	0.0001
Time to modified Aldrete score > 9 (minutes)	6.837 ± 1.052	9.83 ± 0.911	-11.786	0.0001
Sedation score 15 minutes after surgery	2.433 ± 0.192	3.837 ± 0.282	-22.527	0.0001
Sedation score 30 minutes after surgery	2.387 ± 0.185	3.37 ± 0.21	-19.218	0.0003
Sedation score 60 minutes after surgery	1.99 ± 0.3	1.99 ± 0.3	-2.003	0.050
Time to first analgesic request	30.16 ± 2.863	59.48 ± 3.903	-33.180	0.0002

The recovery statistics of both the groups shows statistically significant association with factors such as emergence time, time to modified Alderete score, sedation score at 15 minutes and 30 minutes, and time to first analgesic request in group DEX. Dexmedetomidine has a sedative effect; hence, the emergence time and sedation scores are higher in group DEX than in group E. This difference was found to be highly statistically significant. Table 3 depicts a relatively slower analgesic demand postoperatively and better sedation in GROUP DEX. Mean recovery time was 10.4±1.25 minutes in group E and 11±1.18 minutes in group DEX. Thus, the recovery from hypotension in group E was faster as compared to group DEX, which means that there is hypertension following stoppage of the anesthetic agent; this hampers surgery. However, this difference was not found to be statistically significant. Table 4 shows the distribution of study participants according to surgeon's score.

**Table 4:** Distribution of study participants according to surgeon’s score (n=60)

Surgeon's score	Group				Total		Chi square and p-value
	Esmolol		Dexmedetomidine				
	Number	%	Number	%	Number	%	
1	13	43.33	10	33.33	23	38.33	X^2 ^=0.716, p=0.699
2	15	50.00	17	56.67	32	53.33	
3	2	6.67	3	10.00	5	8.33	
4	0	0	0	0	0	0	
5	0	0	0	0	0	0	
Total	30	100.00	30	100.00	60	100.00	

The operative field conditions were assessed and graded. A grade of 3 or less in both the groups was present, which denoted a highly acceptable surgical field as far as the surgeon was concerned. Patients in group DEX were found to have a less bloody surgical field and received a better score by the surgeon than those in group E. Around four study participants in group E required blood transfusion, whereas the requirement was for two participants in group DEX. This depicted that the requirement of blood transfusion was almost double in group E.

## Discussion

The present study conducted involved 60 patients below 50 years of age planned for FESS. The result of the present study shows that dexmedetomidine was a more efficacious anesthetic agent in achieving hypotensive anesthesia than esmolol while performing FESS. The hemodynamic changes were less profound, with faster reversal to baseline found with group E, but the requirement of longer hypotensive anesthesia and analgesia was seen with dexmedetomidine. Factors such as emergence time, time to modified Aldrete score, sedation score at 15 minutes and 30 minutes, and time to first analgesic request were found to have highly statistically significant association with the anesthetic agents used in the present study, suggesting that dexmedetomidine was more efficacious at providing a bloodless surgical field. The amount of drug required in group DEX is significantly less as compared to group E, and the difference was found to be statistically significant. The dose required to achieve hypotensive anesthesia was also significantly less with the use of dexmedetomidine than that of esmolol. Similar results have been reported by a study conducted in Egypt, where dexmedetomidine was a more efficient drug with better analgesic effect, thereby requiring a delayed demand for analgesia in the postoperative period [6]. Another similar study conducted in Punjab reported that dexmedetomidine and esmolol provided better hemodynamic stability and more bloodless surgical field compared to nitroglycerin during FESS and that dexmedetomidine provided an additional benefit of reducing the analgesic requirements and providing postoperative sedation [10]. In the present study, the blood transfusion requirement was remarkably less with the use of dexmedetomidine, which has not been reported by the aforementioned studies. In a study conducted in China, the researchers tried a combination of esmolol and dexmedetomidine in nasal endoscopic surgery and both dexmedetomidine alone and dexmedetomidine combined with esmolol could effectively control hypotension, but the latter had the advantages of better BP control quality, less pain, and lower probability of adverse reactions such as nausea, vomiting, and bronchospasm, which could improve the safety of nasal endoscopic surgery [11]. It was found that the emergence time in group DEX was found to be higher than that in group E; similar results have been reported in another study conducted in Gujarat, where the emergence time was found to be higher with the use of dexmedetomidine than nitroglycerine [12]. The difference between the emergence time in group DEX and group E was found to be statistically significant. Another study conducted in Tanta University reported that the time of first analgesic request was significantly shorter with esmolol and that the post-operative sedation score at 15 minutes and 30 minutes was found to be longer with the use of dexmedetomidine; these differences were found to be statistically significant, which is similar to the findings of the present study [13]. An interventional study conducted in Jaipur also reported similar results reinforcing the fact that dexmedetomidine provided better hemodynamic stability and comparable operative field visibility during FESS and also provided an additional benefit of reducing the analgesic requirements and providing postoperative sedation [14].

Limitations

The limitation of the present study was that there was no placebo controlled group included as it was not allowed by the ethical committee. Further, esmolol and dexmedetomidine were established based on their known safe optimal doses in the peri-operative context without knowledge of their equipotent doses.

## Conclusions

This study demonstrated that esmolol and dexmedetomidine are safe agents for controlled hypotension and both are effective in providing a bloodless surgical field during FESS. Compared with esmolol, dexmedetomidine offers the advantage of inherent analgesic, sedative, and anesthetic sparing effect. It was also seen that with the use of dexmedetomidine, the need for blood transfusion was remarkably less. The disadvantage of esmolol was the rebound hypertension that occurred when the infusion was turned off, causing rapid perfusion of the surgical field with increased blood loss than dexmedetomidine.

## References

[1] Tan PY, Poopalalingam R (2014). Anaesthetic concerns for functional endoscopic sinus surgery. Proc Singapore Healthcare.

[2] Gohil DH, Patel S (2020). Controlled hypotension for ENT surgery: an observational comparative prospective study between esmolol and sodium nitroprusside. MedPulse Int J Anesth.

[3] Chhabra A, Saini P, Sharma K, Chaudhary N, Singh A, Gupta S (2020). Controlled hypotension for FESS: a randomised double-blinded comparison of magnesium sulphate and dexmedetomidine. Indian J Anaesth.

[4] Gupta S, Sharma KR, Jain D (2005). Airway assessment: predictors of difficult airway. Indian J Anaesth.

[5] Bhavna G, Lalit G (2018). Pulmonary function tests-relevance to anesthesiologists. Anaesth Critic Care Med J.

[6] Shams T, El Bahnasawe NS, Abu-Samra M, El-Masry R (2013). Induced hypotension for functional endoscopic sinus surgery: a comparative study of dexmedetomidine versus esmolol. Saudi J Anaesth.

[7] McGrath B, Chung F (2003). Postoperative recovery and discharge. Anesth Clin North Am.

[8] White PF, Song D (1999). New criteria for fast-tracking after outpatient anesthesia: a comparison with the modified Aldrete's scoring system. Anesth Analg.

[9] Sessler CN, Grap MJ, Ramsay MA (2008). Evaluating and monitoring analgesia and sedation in the intensive care unit. Crit Care.

[10] Bajwa SJ, Kaur J, Kulshrestha A, Haldar R, Sethi R, Singh A (2016). Nitroglycerine, esmolol and dexmedetomidine for induced hypotension during functional endoscopic sinus surgery: A comparative evaluation. J Anaesthesiol Clin Pharmacol.

[11] Liu J, Wang W, Wang J (2020). Dexmedetomidine combined with esmolol controls hypotension in patients undergoing endoscopic surgery. Int J Clin Exp Med.

[12] Shah SM, Reshamwala NS, Raval AS (2020). A comparative study of nitroglycerine and dexmedetomidine for induced hypotension in functional endoscopic sinus surgery. J Evid Based Med Health.

[13] Sharaf MIA, El-nasr LMA, Amin SM, Mohamed RM (2018). Comparison between dexmedetomidine and esmolol for hypotensive anaesthesia during functional endoscopic sinus surgery in children. Med J Cairo Univ.

[14] Usha B, Supriya S, Singh GS, Mamta K (2020). Comparison of hypotensive properties of dexmedetomedine versus nitroglycerine and their effectiveness to provide oligemic surgical field during functional endoscopic sinus surgery: a randomized interventional study. Int J Med Sci Inn Res.

